# Skeletal muscle AMPK is not activated during 2 h of moderate intensity exercise at ∼65% ${\dot{V}}_{O_{2}\mathrm{peak}}$ in endurance trained men

**DOI:** 10.1113/JP277619

**Published:** 2020-07-27

**Authors:** Glenn K. McConell, Glenn D. Wadley, Kieran Le plastrier, Kelly C. Linden

**Affiliations:** ^1^ Institute for Health and Sport Victoria University Melbourne VIC Australia; ^2^ Department of Physiology University of Melbourne Melbourne VIC Australia; ^3^ Institute for Physical Activity and Nutrition School of Exercise and Nutrition Sciences Deakin University Geelong VIC Australia; ^4^ School of Business Western Sydney University Parramatta NSW Australia; ^5^ Faculty of Science Charles Sturt University Albury NSW Australia

**Keywords:** AMPK activity, endurance, exercise, metabolism, trained, training, signalling

## Abstract

**Key points:**

- AMP‐activated protein kinase (AMPK) is considered a major regulator of skeletal muscle metabolism during exercise.
- However, we previously showed that, although AMPK activity increases by 8–10‐fold during ∼120 min of exercise at ∼65% ${\dot{V}}_{O_{2}\mathrm{peak}}$ in untrained individuals, there is no increase in these individuals after only 10 days of exercise training (longitudinal study).
- In a cross‐sectional study, we show that there is also a lack of activation of skeletal muscle AMPK during 120 min of cycling exercise at 65% ${\dot{V}}_{O_{2}\mathrm{peak}}$ in endurance‐trained individuals.
- These findings indicate that AMPK is not an important regulator of exercise metabolism during 120 min of exercise at 65% ${\dot{V}}_{O_{2}\mathrm{peak}}$ in endurance trained men.
- It is important that more energy is directed towards examining other potential regulators of exercise metabolism.

**Abstract:**

AMP‐activated protein kinase (AMPK) is considered a major regulator of skeletal muscle metabolism during exercise. Indeed, AMPK is activated during exercise and activation of AMPK by 5‐aminoimidazole‐4‐carboxyamide‐ribonucleoside (AICAR) increases skeletal muscle glucose uptake and fat oxidation. However, we have previously shown that, although AMPK activity increases by 8–10‐fold during ∼120 min of exercise at ∼65% ${\dot{V}}_{O_{2}\mathrm{peak}}$ in untrained individuals, there is no increase in these individuals after only 10 days of exercise training (longitudinal study). In a cross‐sectional study, we examined whether there is also a lack of activation of skeletal muscle AMPK during 120 min of cycling exercise at 65% ${\dot{V}}_{O_{2}\mathrm{peak}}$ in endurance‐trained individuals. Eleven untrained (UT; ${\dot{V}}_{O_{2}\mathrm{peak}}$ = 37.9 ± 5.6 ml.kg^−1^ min^−1^) and seven endurance trained (ET; ${\dot{V}}_{O_{2}\mathrm{peak}}$ = 61.8 ± 2.2 ml.kg^−1^ min^−1^) males completed 120 min of cycling exercise at 66 ± 4% ${\dot{V}}_{O_{2}\mathrm{peak}}$ (UT: 100 ± 21 W; ET: 190 ± 15 W). Muscle biopsies were obtained at rest and following 30 and 120 min of exercise. Muscle glycogen was significantly (*P* < 0.05) higher before exercise in ET and decreased similarly during exercise in the ET and UT individuals. Exercise significantly increased calculated skeletal muscle free AMP content and more so in the UT individuals. Exercise significantly (*P* < 0.05) increased skeletal muscle AMPK α2 activity (4‐fold), AMPK αThr^172^ phosphorylation (2‐fold) and ACCβ Ser^222^ phosphorylation (2‐fold) in the UT individuals but not in the ET individuals. These findings indicate that AMPK is not an important regulator of exercise metabolism during 120 min of exercise at 65% ${\dot{V}}_{O_{2}\mathrm{peak}}$ in endurance trained men.

## Key points

- AMP‐activated protein kinase (AMPK) is considered a major regulator of skeletal muscle metabolism during exercise.
- However, we previously showed that, although AMPK activity increases by 8–10‐fold during ∼120 min of exercise at ∼65% ${\dot{V}}_{O_{2}\mathrm{peak}}$ in untrained individuals, there is no increase in these individuals after only 10 days of exercise training (longitudinal study).
- In a cross‐sectional study, we show that there is also a lack of activation of skeletal muscle AMPK during 120 min of cycling exercise at 65% ${\dot{V}}_{O_{2}\mathrm{peak}}$ in endurance‐trained individuals.
- These findings indicate that AMPK is not an important regulator of exercise metabolism during 120 min of exercise at 65% ${\dot{V}}_{O_{2}\mathrm{peak}}$ in endurance trained men.
- It is important that more energy is directed towards examining other potential regulators of exercise metabolism.

## Introduction

The signalling events that regulate skeletal muscle exercise metabolism have not been fully elucidated (Richter & Hargreaves, 2013). There is evidence for feed forward (calcium activated CaMK) (Wright *et al*. 2004; Wright *et al*. 2005; Jensen *et al*. 2007; Witczak *et al*. 2010) and feedback (AMP‐activated protein kinase; AMPK) (Hayashi *et al*. 1998; Mu *et al*. 2001; Lee‐Young *et al*. 2009; Abbott *et al*. 2011) regulation being involved, as well as nitric oxide (Balon & Nadler, 1997; Roberts *et al*. 1997; Bradley *et al*. 1999; Inyard *et al*. 2007; Ross *et al*. 2007; Merry *et al*. 2010*a*) and reactive oxygen species production (Toyoda *et al*. 2004; Sandstrom *et al*. 2006; Merry *et al*. 2010*a*), which are increased during contraction (Roberts *et al*. 1999; Reid & Durham, 2002; Linden *et al*. 2011). There is also some evidence that cytosketetal forces during contraction may signal glucose uptake via Rac1 (Sylow *et al*. 2013; Sylow *et al*. 2015).

Skeletal muscle AMPK activity increases during exercise in rodents (Winder & Hardie, 1996; Lee‐Young *et al*. 2009) and humans (Chen *et al*. 2000; Fujii *et al*. 2000; Wojtaszewski *et al*. 2000; Musi *et al*. 2001; Mortensen *et al*. 2013) and, given that activation of AMPK by 5‐aminoimidazole‐4‐carboxyamide‐ribonucleoside (AICAR) increases skeletal muscle glucose uptake and fat oxidation (Merrill *et al*. 1997; Hayashi *et al*. 2000; Jorgensen *et al*. 2004), it has been assumed that activation of AMPK during exercise increase fat and glucose metabolism in humans. However, a number of studies have shown dissociations between activation of AMPK and glucose uptake during muscle contraction in rodents (Jorgensen *et al*. 2004; Fujii *et al*. 2005; Kjobsted *et al*. 2017) and during exercise in humans (Wojtaszewski *et al*. 2000; McConell *et al*. 2005; Mortensen *et al*. 2013). In addition, several studies have shown that fat oxidation increases normally during contraction (Jeppesen *et al*. 2011) and during exercise (Jeppesen *et al*. 2013) in AMPK dominant negative/kinase dead mice.

Further evidence that questions a role for AMPK in glucose and fat metabolism during exercise is that substantial increases in glucose uptake and fat oxidation are seen during low intensity exercise (40–45% ${\dot{V}}_{O_{2}\mathrm{peak}}$) in humans even though AMPK signalling is not increased at such intensities (Wojtaszewski *et al*. 2002; Chen *et al*. 2003). In addition, in a longitudinal study, we showed in previously untrained men that 10 days of exercise training abolishes the ∼10‐fold increase in AMPK α2 activity during exercise at 65% ${\dot{V}}_{O_{2}\mathrm{peak}}$ despite fat oxidation being higher and glucose disposal, although attenuated, still being substantially increased during exercise (McConell *et al*. 2005). We found no relationship between AMPK activation (and AMPK αThr^172^ phosphorylation and ACCβ phosphorylation) and muscle glycogen use, glucose uptake and fat oxidation during exercise (McConell *et al*. 2005). There is no doubt that AMPK activation during exercise is important for post‐exercise adaptations (Winder *et al*. 2000; McGee *et al*. 2003), although these findings imply that AMPK activation during moderate intensity exercise is not necessary for normal increases in glucose uptake and fat oxidation during exercise.

It can be argued that there is less activation of AMPK after short‐term exercise training because there is less of an energy deficit in skeletal muscle during exercise. Indeed, we found this to be the case in humans in our short‐term training study reporting no activation of AMPK during exercise at 65% of pretraining ${\dot{V}}_{O_{2}\mathrm{peak}}$ (McConell *et al*. 2005). The exercise after training was conducted at the pre‐exercise training workload; as such, the exercise was at a little lower relative workload after training (McConell *et al*. 2005). However, it has subsequently been shown in humans that, after 12 weeks of exercise training, there is less activation of skeletal muscle AMPK when the post training exercise was conducted at the same relative intensity of 65% ${\dot{V}}_{O_{2}\mathrm{peak}}$ as the pre‐training exercise (Mortensen *et al*. 2013).

Another way of examining the effect of exercise training on AMPK activation is to conduct a cross‐sectional study comparing endurance trained with untrained individuals exercising at the same relative intensity. Nielsen *et al*. (2003) found similar increases in AMPK α_2_ activity during 20 min of exercise at 80% ${\dot{V}}_{O_{2}\mathrm{peak}}$ in trained compared with untrained individuals. This was surprising because they and others have found less of a skeletal muscle energy imbalance and therefore less of an increase in skeletal muscle AMP and ADP at 80% ${\dot{V}}_{O_{2}\mathrm{peak}}$ in trained compared with untrained men (Baldwin *et al*. 1999; Nielsen *et al*. 2003). It is not known whether skeletal muscle AMPK activity would increase during prolonged moderate intensity exercise in well trained individuals. This is important to determine because, if AMPK does not increase during such exercise, this would imply that AMPK is not important for the regulation of exercise metabolsim during such exercise.

Therefore, the present study aimed to examine whether long‐term endurance exercise trained individuals have an increase in skeletal muscle AMPK during 120 min of exercise at 65% ${\dot{V}}_{O_{2}\mathrm{peak}}$. Based on our findings of no activation at this workload in previously untrained individuals after 10 days of exercise training (McConell *et al*. 2005), we hypothesized that skeletal muscle AMPK would not be activated during 120 min of exercise at 65% ${\dot{V}}_{O_{2}\mathrm{peak}}$ in endurance trained men.

## Methods

### Ethical approval

The present study was approved by the Human Research Ethics Committee of the University of Melbourne (Study number 040090) and conducted in accordance with the *Declaration of Helsinki*, except for registration in a database.

### Subjects

Seven endurance‐trained cyclists and triathletes (26 ± 2 years; 72 ± 4 kg; ${\dot{V}}_{O_{2}\mathrm{peak}}$ = 4.4 ± 0.35 L min^−1^, mean ± SD) and eleven healthy but otherwise untrained (23 ± 3 years; 69 ± 9 kg; ${\dot{V}}_{O_{2}\mathrm{peak}}$ = 2.6 ± 0.4 L min^−1^, mean ± SD) non‐smoker males participated in the study (Table 1). Trained participants cycled on average 300 ± 100 km week^–1^, whereas untrained participants undertook no regular exercise.

**Table 1:** Subject characteristics

Parameter	Untrained	Trained
Age (years)	23 ± 3	26 ± 2
Weight (kg)	69 ± 9	72 ± 4
Height (m)	1.75 ± 0.35	1.76 ± 0.39
BMI (kg m^–2^)	22 ± 2.6	23 ± 0.76
${\dot{V}}_{O_{2}\mathrm{peak}}$ (L min^−1^)	2.60 ± 0.4^*^	4.44 ± 0.35
${\dot{V}}_{O_{2}\mathrm{peak}}$ (ml kg^−1^ min^−1^)	37.9 ± 5.6^*^	61.8 ± 2.2

Values are the mean ± SD, *n* = 11 untrained and 7 exercise trained participants, BMI, body mass index, ${\dot{V}}_{O_{2}}$_,_ oxygen consumption. *Significantly different to corresponding trained value (*P* < 0.05).

### Experimental design

Participants were required to attend the laboratory on three separate occasions. The first visit involved a peak pulmonary oxygen consumption test during cycling (${\dot{V}}_{O_{2}\mathrm{peak}}$), followed 2–3 days later by a 30 min familiarisation ride at a workload calculated from the ${\dot{V}}_{O_{2}\mathrm{peak}}$ test to be ∼65% ${\dot{V}}_{O_{2}\mathrm{peak}}$ to confirm the power output for the experimental trials. Approximately 1 week later, participants returned to the laboratory for an exercise trial, which involved cycling for 120 min at ∼ 65% ${\dot{V}}_{O_{2}\mathrm{peak}}$ (untrained; 100 ± 21 W; trained 190 ± 15) (Table 2).

### Dietary and exercise controls

All participants were asked to refrain from any formal exercise for 48 h prior to the experimental trial to minimise any acute exercise training effects and to avoid drinking alcohol or consumption of caffeine for 24 h prior. To ensure the energy intake was controlled between groups, participants were supplied with a diet to consume over the 24 h prior to each experimental trial containing ∼199 kJ kg^–1^ consisting of ∼65% of carbohydrates, ∼15% proteins and ∼ 20% fats. Participants were instructed to adhere to the diet but to consume water *ad libitum* and to finish the food by 10pm  the evening prior to the experimental trial to enable attending the laboratory in a fasted state.

### Exercise trials

On the morning of the exercise trial, a 22 gauge Teflon catheter (Optiva; Ethicon Endo‐Surgery, Cincinnati, OH, USA) was inserted into an antecubital forearm vein for blood sampling. The exercise protocol consisted of cycling for 120 min at 65% of ${\dot{V}}_{O_{2}\mathrm{peak}}$. Blood was sampled 10 min prior to the commencement of exercise and then every 30 min during exercise for the measurement of plasma glucose, lactate, insulin, glycerol and free fatty acids. Expired air was collected into Douglas bags every 30 min during exercise and heart rate (Polar Favor, Oulu, Finland) was recorded every 30 min during exercise. ${\dot{V}}_{O_{2}}$ and the respiratory exchange ratio were calculated from the expired air samples. Participants received 8 ml kg^−1^ body weight of water at the start of exercise, followed by a further 2 ml kg^−1^ body weight every 15 min of exercise and were fan cooled throughout the trial. At rest and after 30 and 120 min of exercise, muscle was obtained from the vastus lateralis under local anaesthesia, using the percutaneous needle biopsy technique, with suction. Muscle samples were rapidly (8–12 s from stopping exercise) frozen and stored in liquid N_2_ for later analysis of AMPK α1 and α2 activity, AMPK αThr^172^ phosphorylation and ACCβ Ser^222^ phosphorylation and muscle metabolites.

### Analytical techniques

#### Blood

Plasma glucose, lactate (Lowry OH, 1972) and glycerol (Chernick, 1969) were determined using an enzymatic fluorometric procedure, plasma non‐esterified fatty acids (NEFA) by an enzymatic colorimetric method (NEFA‐C test, Wako, Osaka, Japan) and plasma insulin using a human insulin‐specific radioimmunoassay kit (Linco Research, St Charles, MO, USA).

#### Muscle metabolites

A portion of each muscle sample (∼20 mg) was freeze‐dried and subsequently crushed to a powder and any visible connective tissue was removed. The extraction of muscle glycogen commenced by incubating the sample in HCl before being neutralized with NaOH and subsequently analysed for glucosyl units using an enzymatic flurometric method (Passonneau & Lauderdale, 1974). The metabolites (ATP, CrP, Cr and lactate) were extracted firstly with precooled perchloric acid/EDTA before the addition of precooled KHCO_3_ to the supernatant. The metabolites were analysed in triplicate using an enzymatic flurometric method as reported by Harris *et al*. (1974). PCr, Cr and ATP were normalised to the participant's highest total creatine (Cr + CrP). The concentration of ADP (ADP_free_) and AMP (AMP_free_) was calculated based on the near equilibrium nature of the CK and adenylate kinase reactions, respectively. ADP_free_ was calculated from the measured ATP, Cr, PCr levels and the estimated H^+^ concentration, which was calculated from a formula based on the muscle lactate content for dry muscle (Mannion *et al*. 1993). The observed equilibrium constant (*K*_obs_) of 1.66 × 10^9^ was used for creatine kinase (Lawson & Veech, 1979). An estimation of AMP_free_ was calculated from the measured ATP and estimated ADP_free_, using a *K*_obs_ of 1.05 for adenylate kinase (Lawson & Veech, 1979). Estimated ADP_free_ and AMP_free_ were expressed as μmol per kilogram of dry muscle mass (μmol kg^−1^ dry muscle).

#### Immunoblotting

Frozen skeletal muscle was homogenised in ice cold lysis buffer on ice [10 μl mg^–1^ tissue; 50 mm Tris‐HCl, pH 7.5, containing 1 mm EDTA, 1 mm EGTA, 10% v/v glycerol, 1% v/v Triton X‐100, 50 mm NaF, 5 mm Na_4_P_2_O_7_, 1 mm dithiothreitol, 1 mm phenylmethylsulphonyl fluoride, 1 μl ml^−1^ trypsin inhibitor and 5 μl ml^−1^ protease inhibitor cocktail (P8340; Sigma, St Louis, MO, USA)], incubated on ice for 20 min and centrifuged at 16 000 *g* for 20 min at 4°C. The protein concentration of samples was determined using the bicinchoninic acid protein assay (Pierce, Rockford, IL, USA) with BSA as the standard. All primary antibodies were diluted to a final concentration of 1:1000. Phosphospecific antibodies for AMPK αThr^172^ and ACCβ Ser^221^ were purchased from Upstate Biotechnology (Lake Placid, NY, USA; catalogue no. 07–626 and 05–673, respectively). Polyclonal rabbit antibody specific for total AMPK α protein was purchased from Cell Signalling Technology (Beverley, MA, USA; catalogue no. 2532). ACCβ was detected using IRDye^TM^ 800‐labelled streptavidin (Rockland, Gilbertsville, PA, USA; catalogue no. S000‐45).

Skeletal muscle lysates (80 μg) were heated in SDS sample buffer and subjected to SDS‐PAGE. Binding of purified proteins was detected by immunoblotting following an overnight incubation with primary antibody. Membranes were incubated in Odyssey anti‐rabbit IRDye™ 800‐ or anti‐mouse IRDye™ 700‐ labelled secondary antibody (Rockland, Gilbertsville, PA, USA), washed in PBS Tween 20 and were scanned for infra‐red fluorescence using an Odyssey Infrared Imaging System (LI‐COR Biosciences, Lincoln, NE, USA). When both total protein and protein phosphorylation were measured, membranes were probed first for total protein, stripped of antibodies (2% SDS in 25 mm glycine, pH 2.0) and re‐probed with the anti‐phospho antibody. Phosphorylation was expressed relative to the total protein of the specific protein of interest.

#### AMPK activity

Skeletal muscle lysates (50 μg) were combined with 15 μl of protein A sepharose beads (Pierce), bound to either AMPK α1 (raised to the non‐conserved region of the AMPK α1 isoform, amino acid sequence 373–390 of rat AMPK α1) or AMPK α2 (amino acid sequences 351–366 and 490–516 of rat AMPKα2) polyclonal antibodies (a gift from Professor Bruce Kemp, St Vincent's Institute of Medical Research, Fitzroy, VIC, Australia) and incubated for 2 h at 4 °C. Immunocomplexes were washed in lysis buffer containing 0.5 m NaCl and resuspended in 25 μl of 0.05 m Tris buffer (pH 7.5). To commence the assay, 25 μl of reaction mixture containing (final concentrations) 50 mm Tris/HCl, pH 7.5, 0.1 mm EGTA, 0.1% (by vol) 2‐mercaptoethanol, 10 mm magnesium acetate, 0.1 mm [^32^P]ATP (∼200 cpm/pmol; PerkinElmer Life and Analytical Sciences, Boston, MA, USA), 30 μm AMARA peptide (Upstate Biotechnology) and 200 μm AMP was added to each sample at 30°C for 20 min with agitation. Then, 40 μl of each sample was transferred onto P81 chromatography paper and washed 3 × 10 min in 75 mm H_3_PO_4_, once in 100% ethanol, and air dried. P81 paper was then placed in organic scintillation fluid (Opti‐Fluor O; PerkinElmer) and radioactivity was counted on a β counter (PerkinElmer). AMARA peptide has the same AMPK phosphorylation site as ACCβ; therefore, AMPK activities were calculated as units of γ‐[^32^P]‐ATP incorporated into the AMARA peptide [ACCα (73‐87)A^77^] min^−1^ mg^−1^ total protein subjected to immunoprecipitation.

#### Statistical analysis

Data are expressed as the mean ± SD. Untrained and exercise trained group values were compared using a two‐factor (training × time) repeated measures ANOVA and, if there was a significant interaction, the ANOVA was followed by a *post hoc* comparison using a least significant difference test. *P* < 0.05 was considered statistically significant.

## Results

### Subjects

There was no significant difference in age, weight, height or body mass index between the untrained and exercise trained groups (Table 1). However, as expected, ${\dot{V}}_{O_{2}\mathrm{peak}}$ was significantly higher in the endurance trained group (*P* < 0.05) (Table 1) and the trained group cycled on average at almost twice the workload of the untrained group (*P* < 0.05) (Table 2). Importantly, the relative exercise intensity was the same between groups, with no significant difference for ${\dot{V}}_{O_{2}}$ (as a percentage of ${\dot{V}}_{O_{2}\mathrm{peak}}$), heart rate or rating of perceived exhaustion (Table 2).

**Table 2:** Physiological responses during 120 min of steady‐state exercise at ∼ 65% ${\dot{V}}_{O_{2}\mathrm{peak}}$

Parameter	Untrained	Trained
% of ${\dot{V}}_{O_{2}\mathrm{peak}}$ (ml.kg^−1^ min^−1^)	66 ± 4.5	65 ± 1.6
Workload (watts)	100 ± 21^*^	190 ± 15
RER	0.91 ± 0.56	0.91 ± 0.38
RPE	12.4 ± 1.2	11.0 ± 1.5
Heart rate (beats min^–1^)	147 ± 9	143 ± 7

Data are the mean ± SD, *n* = 11 untrained and 7 exercise trained participants. ${\dot{V}}_{O_{2}}$, oxygen consumption, RER, respiratory exchange ratio, RPE, rating of perceived exhaustion.

* Significantly different to corresponding trained value (*P* < 0.05).

### Muscle metabolites

Measured muscle lactate and Cr and estimated ADP_free_, AMP_free_ and the AMP_free_:ATP ratio all increased progressively with exercise in both groups (Table 3). The exercise induced increase in muscle lactate, Cr and AMP_free_ was attenuated in the exercise trained compared to the untrained group (*P* < 0.05) (Table 3). Skeletal muscle AMP_free_ increased (*P* < 0.05) 17‐fold in the untrained group and significantly (*P* < 0.05) less (5‐fold) in the endurance trained group following 120 min of exercise and the muscle lactate concentration increased by 5.9‐fold in the untrained and 3.1‐fold in the well trained individuals (*P* < 0.05) (Table 3). PCr and the PCr:(PCr+Cr) ratio decreased progressively in both groups, and this decrease was greater in untrained subjects (∼26% decrease in untrained, ∼44% decrease in endurance trained groups, *P* < 0.05) (Table 3). There was no difference in measured ATP levels during exercise or between groups (*P* > 0.05) (Table 3).

**Table 3:** Measured and calculated muscle metabolites at rest (0 min), and after 30 min and 120 min of steady‐state exercise at ∼ 65% of ${\dot{V}}_{O_{2}\mathrm{peak}}$

Metabolite			0 min	30 min	120 min
Lactate	Untrained	^§^	4.6 ± 1.2	11.9 ± 3.4	27.5 ± 11.6^*^
(mmol kg^−1^ dm)	Trained	^§‡^	3.8 ± 2.1	8.9 ± 3.6	11.6 ± 4.9
PCr	Untrained	^§^	70.1 ± 15.5	46.1 ± 12.8	42.9 ± 11.5
(mmol kg^−1^ dm)	Trained	^§‡^	78.9 ± 13.7	61.5 ± 17.0	56.6 ± 13.8
Cr	Untrained	^§^	38.5 ± 7.0	61.4 ± 18.3	69.7 ± 17.6
(mmol kg^−1^ dm)	Trained	^§‡^	39.1 ± 6.1	55.1 ± 11.1	58.8 ± 15.9
PCr:(PCr + Cr)	Untrained	^§^	0.64 ± 0.07	0.44 ± 0.12	0.37 ± 0.13
	Trained	^§‡^	0.66 ± 0.07	0.52 ± 0.12	0.50 ± 0.12
ATP	Untrained		24.3 ± 2.2	24.1 ± 2.5	24.3 ± 3.5
(mmol kg^−1^ dm)	Trained		24.4 ± 4.1	26.5 ± 6.2	24.1 ± 3.0
ADP_free_	Untrained	^§^	131 ± 36	338 ± 239	409 ± 199
(μmol kg^−1^ dm)	Trained	^§^	109 ± 20	228 ± 66	248 ± 117
AMP_free_	Untrained	^§^	0.8 ± 0.5	2.3 ± 0.8	13.5 ± 4.1^*^
(μmol kg^−1^ dm)	Trained	^§‡^	0.6 ± 0.3	2.4 ± 1.5	3.2 ± 2.6
AMP_free_:ATP	Untrained	^§^	0.04 ± 0.02	0.08 ± 0.03	0.56 ± 0.17
	Trained	^§‡^	0.03 ± 0.02	0.10 ± 0.07	0.13 ± 0.09

Values are the mean ± SD, *n* = 11 untrained and 7 exercise trained participants. PCr, creatine phosphate; Cr, creatine; ADP_free_, free adenosine diphosphate; AMP_free_, free adenosine monophosphate, dm; dry muscle. ^*^Significantly different to corresponding trained value. §Main effect for time. ‡Main effect for untrained compared to trained (*P* < 0.05).

**Figure 1:**
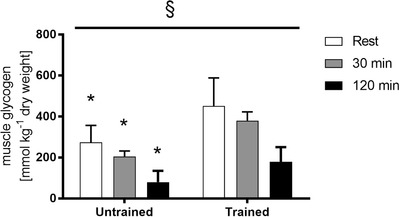
Muscle glycogen measurements in muscle samples Muscle glycogen measured from muscle samples obtained before exercise (rest), after 30 min and immediately following 120 min of steady‐state exercise at ∼65% ${\dot{V}}_{O_{2}\mathrm{peak}}$ in untrained and exercise trained participants. Data are the mean ± SD (*n* = 11 untrained, 7 trained. ^*^Significantly different to corresponding trained value (*P* < 0.05). §Main effect for time (*P* < 0.05).

Resting muscle glycogen levels were 93% higher (*P* < 0.05) in the exercise trained group, and remained higher than the untrained values at 30 min (130%, *P* < 0.05) and 120 min of exercise (440%, *P* < 0.05) (Fig. 1). However, net glycogen utilization during exercise was similar between the two groups.

### Plasma glucose lactate, insulin, glycerol and NEFA

**Figure 2:**
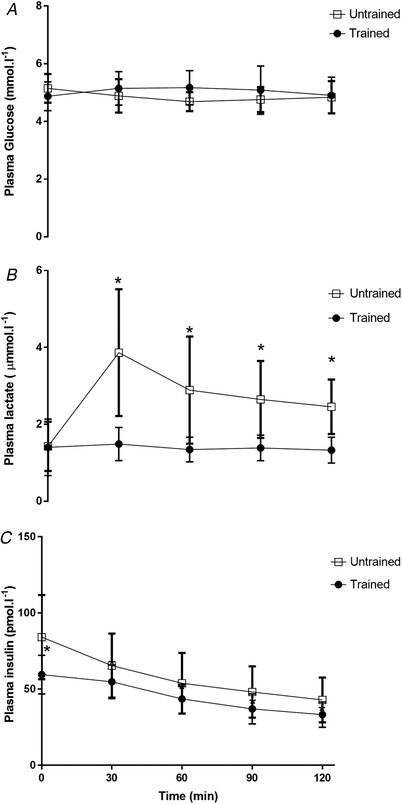
Plasma glucose, lactate and insulin concentrations Plasma glucose (*A*), plasma lactate (*B*) and plasma insulin (*C*) concentrations at rest and during 120 min of steady‐state exercise at ∼65% ${\dot{V}}_{O_{2}\mathrm{peak}}$ in untrained and exercise trained participants. Data are the mean ± SD, *n* = 11 untrained, 7 trained. ^*^Significantly different from corresponding trained value (*P* < 0.05).

**Figure 3:**
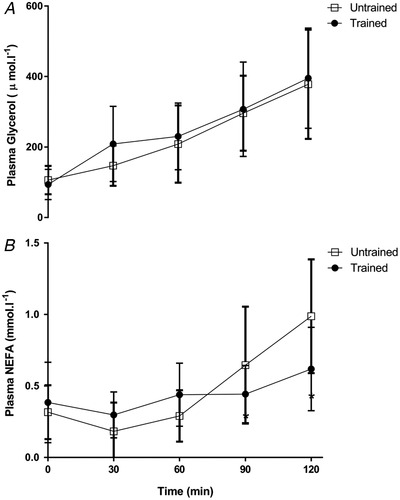
Plasma glycerol and nonesterified fatty acid concentrations Plasma glycerol (*A*) and plasma nonesterified fatty acid (NEFA) (*B*) concentrations at rest and during 120 min of steady‐state exercise at ∼65% ${\dot{V}}_{O_{2}\mathrm{peak}}$ in untrained and exercise trained participants. Data are the mean ± SD, *n* = 11 untrained, 7 trained.

Plasma glucose concentration remained at a similar level during exercise in untrained and endurance trained subjects (*P* > 0.05) (Fig. 2*A*). Plasma lactate was similar at rest in the two groups; however, it was elevated by ∼110% in the untrained group during exercise, which was significantly higher than the trained group (*P* < 0.05) (Fig. 2*B*). Plasma insulin decreased progressively throughout exercise in both groups; however, fasting plasma insulin was 40% higher in the untrained compared to the trained group at rest, and remained ∼25% higher than the trained values throughout 120 min of exercise (*P* < 0.05) (Fig. 2*C*). Both plasma glycerol and NEFA increased progressively throughout exercise in both groups; however, NEFA levels were attenuated in the exercise trained group at 90 and 120 min (*P* < 0.05) (Fig. 3).

### AMPK signalling

**Figure 4:**
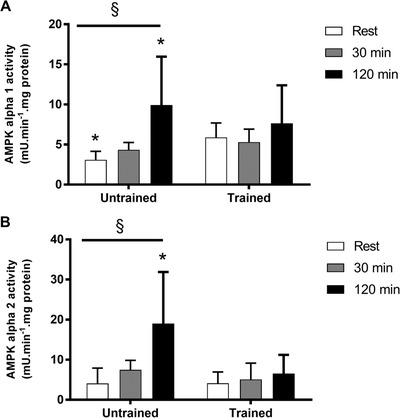
Skeletal muscle AMPK α1 and α2 activity AMPK α1 (*A*) and α2 activity (*B*). Muscle samples were obtained before exercise (rest), after 30 min and immediately following 120 min of steady‐state exercise at ∼65% ${\dot{V}}_{O_{2}\mathrm{peak}}$ in untrained and exercise trained participants. Data are the mean ± SD, *n* = 11 untrained, 7 trained. ^*^Significantly different from corresponding trained value (*P* < 0.05). ^§^Main effect for time (*P* < 0.05).

**Figure 5:**
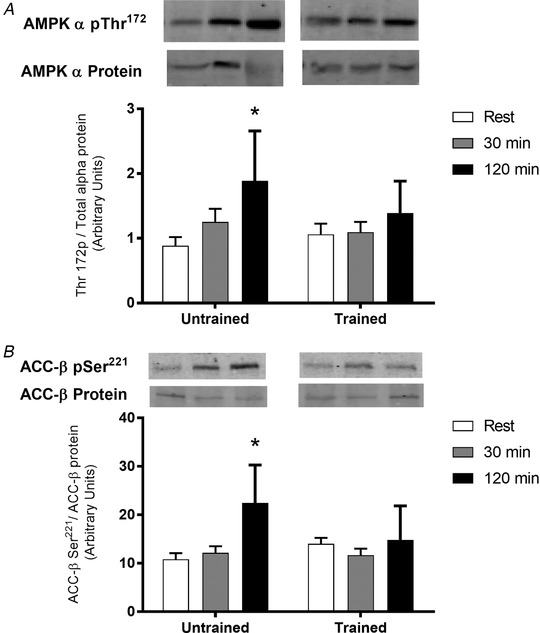
Skeletal muscle AMPK αThr^172^ and ACC‐βSer^221^ phosphorylation Representative immunoblot of AMPK αThr^172^ phosphorylation (*A*) measured using a phosphospecific antibody for AMPK αThr^172^, normalized to total AMPK α protein; and ACC‐βSer^221^ phosphorylation (*B*) measured using a phosphospecific antibody specific to ACC‐βSer^221^, normalized to total ACC‐β. Muscle samples were obtained before exercise (rest), after 30 min and immediately following 120 min of steady‐state exercise at ∼65% ${\dot{V}}_{O_{2}\mathrm{peak}}$ in untrained and exercise trained participants. Samples of pAMPK or AMPK for each participant were run on the same western blot. The representative western blots for pAMPK or AMPK shown are from the same membrane but are rearranged for clarity. Data are the mean ± SD, *n* = 11 untrained, 7 trained. ^*^Significantly different from corresponding trained value (*P* < 0.05). §Main effect for time (*P* < 0.05).

Basal AMPK α1 activity was 90% higher (*P* < 0.05) in the exercise trained group, whereas there was no difference in basal AMPK α2 activity between the two groups (Fig. 4). AMPK α1 activity increased by 220% and AMPK α2 activity increased by 370% during exercise in the untrained group at 120 min (*P* < 0.05). However, neither AMPK α1, nor AMPK α2 activity increased during exercise in the endurance trained group (*P* > 0.05) (Fig. 4). AMPK αThr^172^ (115%, *P* < 0.05) phosphorylation and ACCβ Ser^222^ phosphorylation (100%, *P* < 0.05) increased with exercise in the untrained group after 120 min of exercise (Fig. 5). However, there was no significant increase in AMPK αThr^172^ phosphorylation or ACCβ Ser^222^ phosphorylation during exercise in the trained group (*P* > 0.05) (Fig. 5).

## Discussion

The results of the present study show that AMPK activity is not increased during prolonged steady‐state, moderate intensity exercise in endurance trained individuals. Indeed, AMPK α1 and α2 activity was significantly elevated following 120 min of exercise in the untrained group (220% and 370%, respectively), whereas no increase in AMPK activity was observed during exercise in the trained participants (Fig. 4). Given that there is a substantial amount of glucose and fat oxidised during 120 min of exercise at 65% ${\dot{V}}_{O_{2}\mathrm{peak}}$ in endurance trained individuals (Romijn *et al*. 1993; van Loon *et al*. 2001), these results indicate that AMPK activation is not important for exercise metabolism under these circumstances.

Almost every study investigating AMPK states in their Introduction that AMPK regulates glucose uptake and fat oxidation during exercise, despite much evidence to the contrary. Indeed, our current findings are consistent with our previous results indicating that, after 10 days of exercise training, there was no increase from rest in skeletal muscle AMPK activity, AMPK αThr^172^ phosphorylation or ACCβ phosphorylation during 120 min of exercise at 65% of pre‐training ${\dot{V}}_{O_{2}\mathrm{peak}}$ despite substantial glucose disposal and higher fat oxidation during exercise after (compared to before) exercise training (McConell *et al*. 2005). Other studies similarly report in humans that skeletal muscle AMPK activation is barely increased during exercise at 65% ${\dot{V}}_{O_{2}\mathrm{peak}}$ after 12 weeks of exercise training (Mortensen *et al*. 2013). Although not universally the case (Mu *et al*. 2001; Sakamoto *et al*. 2005; Lee‐Young *et al*. 2009), many rodent studies have found that there are normal increases in glucose uptake and fat oxidation during contraction or during exercise in AMPK kinase dead or AMPK knockout mice (Jorgensen *et al*. 2004; Fujii *et al*. 2005; Merry *et al*. 2010*b*; Jeppesen *et al*. 2011; Jeppesen *et al*. 2013; Kjobsted *et al*. 2017).

Unlike the present study, it was reported previously that skeletal muscle AMPK activity increases similarly in untrained and well trained people cycling for 20 min at 80% ${\dot{V}}_{O_{2}\mathrm{peak}}$ (Nielsen *et al*. 2003). In addition, Clark *et al*. (2004) found AMPK activity increased similarly during repeated bouts of exercise at 85% ${\dot{V}}_{O_{2}\mathrm{peak}}$ after exercise training despite evidence of lower energy deficit after the exercise training. In the present study, exercise was performed at ∼65% of ${\dot{V}}_{O_{2}\mathrm{peak}}$ (untrained 66%; trained 65%) (Table 2); some may argue that this workload is quite easy for trained individuals and so no increase in AMPK activation would be expected. However, because there are 2–3‐fold increases from rest in whole‐body glucose uptake and fat oxidation during moderate intensity exercise at ∼65% ${\dot{V}}_{O_{2}\mathrm{peak}}$ in endurance trained individuals (Romijn *et al*. 1993; van Loon *et al*. 2001), factors that are important to mediating exercise metabolism should be enhanced at these workloads. Therefore, activation of skeletal muscle AMPK is probably not necessary for increases in glucose uptake and fat oxidation during moderate intensity exercise in humans. These findings are also supported by other human studies that found dissociations between AMPK activation and glucose uptake and fat oxidation during prolonged exercise at 40–45% ${\dot{V}}_{O_{2}\max}$ (Chen *et al*. 2000; Wojtaszewski *et al*. 2002).

AMPK activity is increased by upstream kinase phosphorylation and allosterically by increases in AMP (Hardie, 2004; Sakamoto *et al*. 2005). In addition, ADP increases AMPK activity by preventing dephosphorylation of AMPK (Xiao *et al*. 2011). Exercise increases skeletal muscle free ADP and AMP (McConell *et al*. 1999) and exercise training attenuates the normal increases in ADP and AMP during exercise compared to untrained muscle (Gollnick & Hermansen, 1973). Indeed, in the present study, we report an attenuated increase in calculated AMP and ADP during exercise in the well trained compared to the untrained participants (Table 3) and thus would have expected a similar attenuated response in AMPK activity. In the present study, despite the five‐fold increase in AMP_free_ during exercise in the trained individuals AMPK activity was not increased from rest. Future studies should investigate whether there is a reduction in AMPK sensitivity to AMP and ADP during moderate intensity exercise after exercise training. This could be carried out in highly trained endurance subjects by using exercise intensity to titrate the free AMP levels to compare the threshold for AMPK activation during exercise in endurance trained *vs*. untrained individuals.

It is possible that AMPK activation during exercise in the trained individuals was restrained by the higher muscle glycogen since muscle glycogen content has also been implicated as a potential regulator of AMPK activation during exercise. Indeed, the β subunit contains a glycogen binding domain that associates with glycogen in cell free assays (Hudson *et al*. 2003; Polekhina *et al*. 2003). High muscle glycogen has been shown to inhibit contraction induced AMPK activation without influencing glucose uptake in rats (Derave *et al*. 2000). However, there is evidence to suggest that AMPK does not associate with glycogen *in vivo* (Viollet *et al*. 2003; Parker *et al*. 2007). Indeed our laboratory has shown that muscle glycogen is not responsible for abolished AMPK activity during exercise in humans. Following short‐term exercise training, there is no increase in AMPK activity during exercise commenced with normal or high glycogen levels (McConell *et al*. 2005). In the present study, muscle glycogen content was 93% higher in the trained subjects at rest, and remained higher than untrained values throughout the exercise trial (Fig. 1). However, given the findings of our short‐term exercise training study (McConell *et al*. 2005), as well as other evidence of a lack of importance of AMPK for in vivo muscle glycogen content (Viollet *et al*. 2003), the higher muscle glycogen content in the endurance trained individuals was probably not responsible for the abolished increase in AMPK activity during exercise.

It is not clear why activation of AMPK αThr^172^ phosphorylation and AMPK activity did not occur during exercise in the exercise trained individuals; however, there is some evidence to suggest that training is associated with changes in the regulation of upstream AMPKK(s). For example, endurance training in rats decreases basal LKB1 activity (Hurst *et al*. 2005) and decreases AMPK activity during exercise (Durante *et al*. 2002). It is also not known what effect exercise training has on PP2C activity, and it may be that increases in PP2C activity following training are preventing AMPK αThr^172^ phosphorylation and increases in AMPK activity during exercise. PP2C has previously been shown to inhibit the activity of both AMPK α1 and α2 isoforms (Davies *et al*. 1995); however, further studies are required to determine whether changes in PP2C activity occur following exercise training.

In summary, the present study found that, unlike untrained individuals, endurance trained men have no increase in skeletal muscle AMPK activity, AMPK phosphorylation or ACC phosphorylation during 120 min of cycling exercise at ∼65% ${\dot{V}}_{O_{2}\mathrm{peak}}$. This finding is consistent with results following short‐term exercise training where no increase in AMPK activation during moderate intensity exercise is also observed (McConell *et al*. 2005). Given that skeletal muscle AMPK is not activated but fat oxidation and glucose uptake are substantial during moderate intensity exercise after exercise training, these results indicate that AMPK does not regulate metabolism under these circumstances.

## Additional information

### Competing interests

The authors declare that they have no competing interests.

### Funding

This work was supported by a grant from the National Health and Medical Research Council (NHMRC) of Australia (GKM: 237002).

### Author contributions

GKM, KCL and GDW designed the study. GKM, KCL conducted the exercise tests. GKM, KCL, GDW and KLP conducted the experiments. KCL and GDW conducted the analysis. GKM and KCL drafted the manuscript. All authors revised the manuscript critically.

## Supporting information


**Statistical Summary Document**

## Data Availability

The data that support the findings of this study are available from the corresponding author upon reasonable request.
